# Microfluidic Cultivation and Laser Tweezers Raman Spectroscopy of *E. coli* under Antibiotic Stress

**DOI:** 10.3390/s18051623

**Published:** 2018-05-18

**Authors:** Zdeněk Pilát, Silvie Bernatová, Jan Ježek, Johanna Kirchhoff, Astrid Tannert, Ute Neugebauer, Ota Samek, Pavel Zemánek

**Affiliations:** 1 Institute of Scientific Instruments (ISI), Czech Academy of Sciences, v.v.i., Kralovopolska 147, 612 64 Brno, Czech Republic; berns@isibrno.cz (S.B.); jezek@isibrno.cz (J.J.); osamek@isibrno.cz (O.S.); pavlik@isibrno.cz (P.Z.); 2 Center for Sepsis Control and Care (CSCC), Jena University Hospital, Am Klinikum 1, D-07747 Jena, Germany; Johanna.Kirchhoff@med.uni-jena.de (J.K.); Astrid.Tannert@med.uni-jena.de (A.T.); ute.neugebauer@med.uni-jena.de (U.N.); 3 Institute of Physical Chemistry, Friedrich Schiller University Jena, Helmholtzweg 4, D-07743 Jena, Germany; 4 Leibniz Institute of Photonic Technology (Leibniz IPHT), Albert-Einstein-Str. 9, D-07745 Jena, Germany

**Keywords:** Raman micro-spectroscopy, optical tweezers, opto-fluidics, *E. coli*, antibiotics

## Abstract

Analyzing the cells in various body fluids can greatly deepen the understanding of the mechanisms governing the cellular physiology. Due to the variability of physiological and metabolic states, it is important to be able to perform such studies on individual cells. Therefore, we developed an optofluidic system in which we precisely manipulated and monitored individual cells of *Escherichia coli*. We tested optical micromanipulation in a microfluidic chamber chip by transferring individual bacteria into the chambers. We then subjected the cells in the chambers to antibiotic cefotaxime and we observed the changes by using time-lapse microscopy. Separately, we used laser tweezers Raman spectroscopy (LTRS) in a different micro-chamber chip to manipulate and analyze individual cefotaxime-treated *E. coli* cells. Additionally, we performed conventional Raman micro-spectroscopic measurements of *E. coli* cells in a micro-chamber. We found observable changes in the cellular morphology (cell elongation) and in Raman spectra, which were consistent with other recently published observations. The principal component analysis (PCA) of Raman data distinguished between the cefotaxime treated cells and control. We tested the capabilities of the optofluidic system and found it to be a reliable and versatile solution for this class of microbiological experiments.

## 1. Introduction

Raman spectroscopy combined with laser tweezers (LTRS) and a microfluidic chip that allows compartmentalization of a few individual cells and highly controlled exchange of the cell suspension fluids can form the basis of a system for cell analysis, micromanipulation, and sorting [1,2]. Raman spectroscopy is an analytical method that is based on detecting the vibrations of chemical bonds of molecules present in cells and nature in general, which makes it ideal for metabolomic analysis [3,4] and Raman fingerprinting [5,6,7]. After acquiring the spectrum from optically trapped cell, the data is analyzed and the cell can be subsequently sorted by an active micromanipulation with the optical trap [8,9]. Properly implemented cell micromanipulation and LTRS is a completely non-invasive process and the cells can be used for further cultivation and analysis [10,11]. Furthermore, LTRS implemented in the microfluidic chip can serve to study the dynamics of the response of an individual cell to a controlled external stimulus or stress factor. This can be achieved by creating a concentration gradient and moving the studied cells into different compartments on the chip containing different antibiotic concentration and monitoring their response using Raman spectroscopy [12].

New methods to characterize the antibiotic susceptibility of bacterial pathogens in short times are of utmost importance. In times of rising antibiotic resistances, the known resistance pattern of a pathogen helps the treating physician prescribe the right antibiotic therapy in time. Established antibiotic susceptibility testing in the clinical routine is based on time-consuming cultivation and the result is usually not obtained before one or sometimes even after two days. Emerging alternative methods such as new methods based on polymerase chain reaction (PCR) are much faster, but also very costly. Raman spectroscopy as a label-free and non-invasive method holds high potential to advance fast antibiotic susceptibility testing. It was already shown that successful antibiotic-bacteria interaction can be probed after half an hour only [13], which can be utilized in a fast antibiotic susceptibility testing within only 3.5 h [14,15]. Furthermore, it can also be used to quantitatively determine the minimal inhibitory concentration [16].

The ultimate application of this promising analytical method to body fluids requires advanced microfluidic technology. Different approaches to Raman spectroscopy were already implemented into microfluidic devices. Dielectrophoresis [14,17] as well as centrifugal force [18] could successfully be applied to enrich the bacteria from urine samples. LTRS systems combined with microfluidic techniques offer the potential difference to selectively remove cells from body liquids, which are not targeted for analysis. We have developed several solutions in the area combining lasers and microfluidic environment [19,20]. The chamber design was found to be quite successful for optical trapping experiments involving yeast cells [19] and, currently, we use it for experiments with *E. coli*. We aim to effectively combine microfluidics with our expertise in Raman analysis of bacteria and cells in general [21,22,23,24,25,26,27].

Microfluidic chips with cell incubation micro-chambers fabricated in ISI were used for our experiments. The design was optimized based on the previous experiences from their use and the experimental needs. We generated a laminar flow of cultivation medium in the chip. We loaded the bacterial cells and then we used optical tweezers to transport these cells into the micro-chambers. In case of flow-through micro-chambers, the cells were introduced directly. During the experiment, the cells were placed in these dedicated incubation micro-chambers to prevent them from moving away with the cultivation medium flow and to allow undisturbed acquisition of time-lapse images or Raman spectra. After the antibiotic was introduced into the medium flow, within a few seconds it freely diffused into the micro-chambers. Therefore, the concentration of the applied stress factor (antibiotic) at the cell location and the time of exposure of the cells to the stimulus was precisely defined. This on-chip introduction of antibiotics was used for time-lapse imaging experiments concerning filamentous growth of the bacteria under the influence of an antibiotic. In our experiments with LTRS and Raman with regard to the cell response to the antibiotic, we preferred using bacterial cells pre-treated with antibiotics and washed with buffer off-chip prior to introducing them into the micro-chambers, concentrating the cells, and eliminating the background from the cultivation medium by the process. We used 785 nm excitation wavelength for LTRS and 532 nm excitation wavelength for Raman spectroscopy. We compared the results and established the common presence of several annotated Raman peaks specific for *E. coli* in both experimental datasets. In both cases, we detected the changes in Raman spectra of the bacterial cells in response to the antibiotic treatment by principal component analysis (PCA).

## 2. Materials and Methods

### 2.1. Optofluidic System

**Figure 1:**
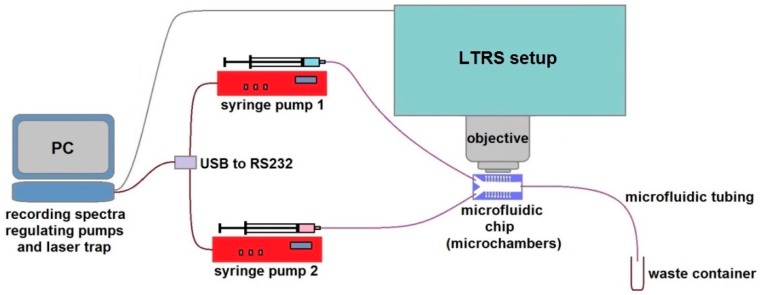
An opto-fluidic system for studying individual living bacteria by laser trapping—Raman spectroscopy (LTRS) in the microfluidic environment. The microfluidic chip with micro-chambers under the microscope objective of the LTRS system is interconnected with the syringe pumps that supply the cultivation medium and the tested antibiotic solution. The pumps and the LTRS system are regulated from dedicated software on a PC.

The layout of our specialized system for LTRS in microfluidic chip with micro-chambers is schematically depicted in Figure 1. We used it in combination with computer programmable syringe pumps (1–5 pumping units according to needs), which supply different liquids into the microfluidic micro-chamber chip such as different media, buffers, antibiotics solutions, and inoculum. The microfluidic part of the system consisted of syringe pumps (NE1001, New Era Pump Systems, Inc., Farmingdale, NY, USA), 1 mL glass syringes (Hamilton, Bonaduz, Switzerland), luer-lock connectors (IDEX Health & Science LLC, Oak Harbor, WA, USA), and microfluidic tubing from the same manufacturer (PEEK, internal diameter 360 μm), which connected the chip to the syringe on one end of the main channel and to a waste container on the opposite end. In all the experiments, the flow rate of the cultivation medium was set to 100 μL/h.

### 2.2. LTRS System

**Figure 2:**
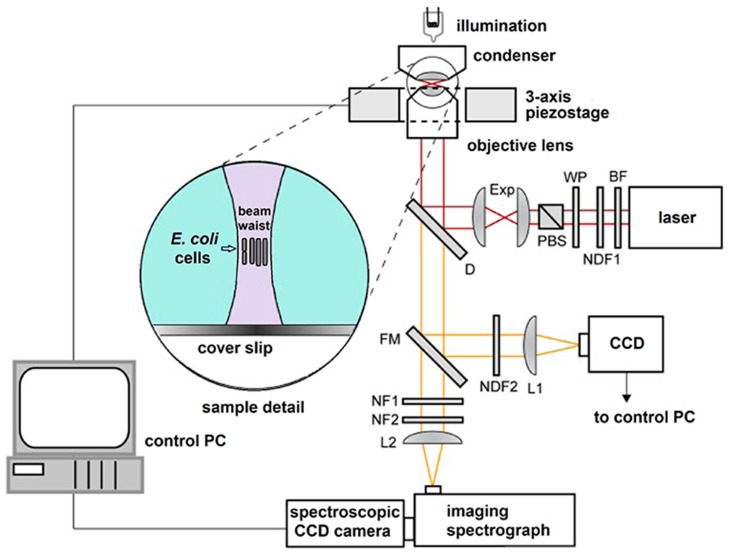
Schematic diagram of the LTRS setup where the same laser beam is used for optical trapping and Raman analysis. BF–band pass filter, D—dichroic mirror, Exp—beam expander, FM—flipping mirror, L1,2—lenses, NDF1,2—neutral density filters, NF1,2—edge filters, PBS—polarizing beam splitter, WP—lambda-half wave plate. Inset shows the detail of optically trapped bacteria near the focus of the laser beam. See details in the main text.

Main element of our opto-fluidic setup is the homemade laser tweezers – Raman spectroscopy (LTRS) system. This system was a modified version of the setup used by Bernatová et al. [24]. The schematic diagram of the LTRS setup is on Figure 2. It combines a Raman micro-spectrometer with optical tweezers [28,29], which provides spatial confinement of individual bacterial cells during Raman spectrum acquisition. The same laser beam is used for optical trapping and Raman spectroscopy. The output beam from a laser (output power ~0.5 W, λ = 785 nm, Sacher Lasertechnik GmbH, Marburg, Germany) was delivered to the setup by an optical fiber and its diameter was expanded three times by an external telescope (not shown in Figure 2). From the telescope the beam passed through a bandpass filter BF (transmission bandwidth 3 nm centered on 785 nm; MaxLine LL01-785, Semrock, Rochester, NY, USA) to eliminate unwanted laser wavelengths. The power of the laser beam for Raman spectroscopy was roughly adjusted by a neutral density filter NDF1 and the fine setting was done by a combination of a λ/2 wave plate WP and a polarizing beam splitter PBS. Beam diameter was further enlarged two times by the beam expander Exp. The laser beam was coupled to the microscope frame via a dichroic mirror D (LPD01-785RS, Semrock) and focused on the specimen with a water-immersion objective lens (UPLSAPO 60×, NA 1.20, Olympus, Tokyo, Japan). The maximal available laser power at the specimen plane was approximately 150 mW. The objective was mounted on a custom-made aluminum frame that also provided a stable support for the sample illumination path and three-axis piezo-driven stage (P-517.3CL, Physik Instrumente, Karlsruhe, Germany) for positioning the sample relative to the beam focus. The Raman scattered light from the trapped microorganism was collected by the same water-immersion objective and was focused by a lens L2 on the entrance slit of an imaging spectrograph (focal length 300 mm, f/3.9, 600 gr/mm diffraction grating, SpectraPro 2300i, PI Acton, Acton, MA, USA), imaged on the chip of a high-sensitivity liquid-nitrogen-cooled spectroscopic CCD camera (Spec-10:100BR/LN, Princeton Instruments, Acton, MA, USA), and recorded using the camera control software (WinSpec, Acton, MA, USA). Rayleigh scattered light at the laser wavelength was blocked by two edge filters NF1 (ZX000626, Iridian, Ottawa, ON, Canada) and NF2 (LP02-785RS, Semrock) and did not enter the spectrograph.

### 2.3. Microfluidic Chips

A crucial element of the optofluidic system is the microfluidic chip. Our microfluidic chips were fabricated from poly(dimethyl)siloxane (PDMS) by conventional soft lithography using master stamps based on negative SU-8 epoxy photo resistance deposited on a silicon substrate [19,30]. SU-8 was spin-coated on the silicon wafer, illuminated by a UV lamp through a mask, and developed. The masks for photolithographic patterning of SU-8 were fabricated by ink-jet printing on a transparent foil by a specialized company (Gatema, Brno, Czech Republic). The PDMS mixture (base to curing agent ratio of 10:1) was then poured into a mold formed by the SU-8 master stamp on Si wafer at the bottom and a square frame machined from polycarbonate. After curing, the resultant PDMS device was peeled off from the mold and attached to a glass slide using standard oxygen plasma treatment.

**Figure 3:**
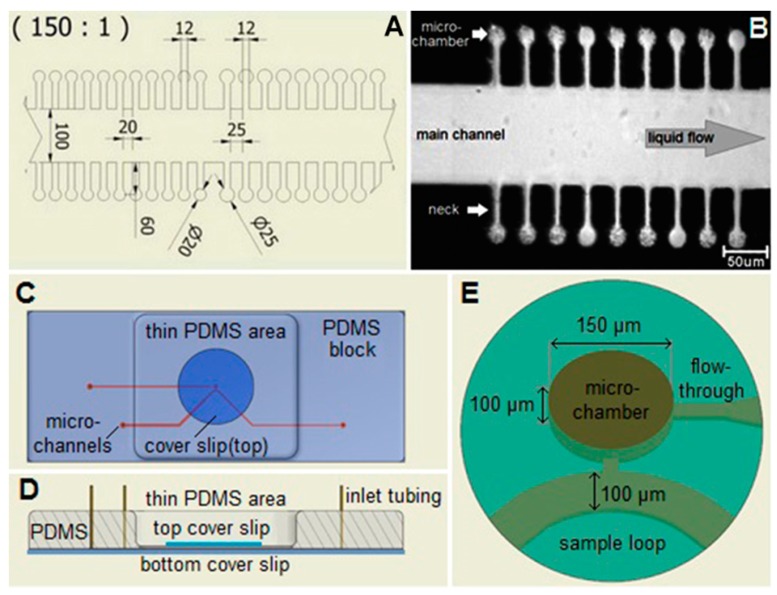
Microfluidic chamber chips used for *E. coli* cultivation, Raman spectroscopy, and optical trapping experiments. (**A**) A detail of the central part of the chip for optical trapping and cell cultivation experiments (dimensions in µm); (**B**) A microscope image of individual micro-chambers in the chip and the adjacent main channel. The main channel in the center is connected with narrow necks to the micro-chambers. *E. coli* cells are present in most of the chambers. They appear dark and dot-shaped or rod-shaped depending on their positions; (**C**) Micro-chamber chip for LTRS and Raman spectroscopy, top view; (**D**) Side view, cross section. (**E**) Detail of the central part with the micro-chamber. The main channel delivers fresh culture medium to the cells in the chambers. The nutrients from the medium and the products of bacterial metabolism diffuse through the neck in and out of the micro-chamber.

The layout of microfluidic chips used in the experiments was previously employed [19] and is apparent from Figure 3. For experiments with optical trapping and cell cultivation, the individual sample chambers of cylindrical shape (diameter 20 μm or 25 μm) were connected to the wide main microfluidic channel (width 100 μm) by side channels of width 12 μm and length 60 μm. Height of all chambers and channels in the chip was 20 μm. Such configuration ensured that the cells were held close to the focal plane of the microscope and could not escape easily from the chambers only due to their diffusion. On the other hand, the length of the side channels was sufficiently short to permit diffusion-mediated replenishment of nutrients in the chambers during the course of the experiment. For experiments with Raman spectroscopy and LTRS, we used larger cylindrical flow-through chambers with a diameter of 150 μm and a height around 100 μm, which were enclosed by a glass cover slip from both the top and bottom side. This allowed acquisition of spectra without interfering Raman signals of PDMS with more maneuvering space for LTRS and lower Raman background from glass.

### 2.4. Bacterial Samples: Strain and Growth Condition

In this study, the patient isolate *E. coli* 683 was used. This strain originated from the blood of a sepsis patient and is part of the strain collection at the Pathogen Biobank at the Institute of Medical Microbiology and the Center for Sepsis Control and Care of Jena University Hospital. Casein soya (CASO) medium (Sigma-Aldrich, St. Louis, MO, USA, sterilized by autoclaving for 15 minutes at 120 °C) was used for cultivation. A sample of bacteria was cultivated on a CASO agar plate and was then transferred to liquid medium and incubated with shaking at 37 °C for 60 min. before injection into the chip or off-chip cultivation with cefotaxime (2 mg/L in CASO medium). The cell count of the injected culture was in the order of 10^6^ cells/mL. Small variations in the cell count of the injected culture had no influence on the experiment. The volume of injected bacterial sample was in the order of 10 µL or less. The proportion of the sample available for micromanipulation, LTRS, or Raman spectroscopy was considerably smaller depending on the field of view and the excitation laser beam parameters, respectively.

### 2.5. Optical TRAPPING Procedure for Time-Lapse Imaging Experiments

The procedure for optical trapping experiments with bacterial cells, which are similar to our previous experiments [19,31], follows. First, the cell culture suspended in the CASO medium was introduced into the main microfluidic channel. Subsequently, all cells studied in a single experimental run were placed one-by-one into adjacent micro-chambers using low-power optical tweezers. In order to minimize the impact of optical trapping on the cells, we adjusted the laser power near the minimal effective trapping power (approx. 10 mW). In addition, this initial optical manipulation was carried out as quickly as possible in less than 10 s. All analyzed cells were well isolated from the bulk of the cell culture. CASO medium with cefotaxime (2 mg/L) was introduced into the microfluidic chip after the experiment started. The cells from these experiments were used for time-lapse cultivation imaging experiments.

### 2.6. LTRS Protocol for Raman Characterization of E. coli with 785 nm Excitation

*E. coli* cells were cultivated for 2 h with shaking at 37 °C in CASO broth with (+) and without (−) 2 mg/L cefotaxime added to the medium. The cells were centrifuged for 4 min at 5000× *g*, the supernatant was discarded, and the pellet washed with 1 mL of cold PBS three times before the LTRS measurement in order to remove any interfering Raman signal from the cultivation medium. Both the optical trapping and Raman excitation was realized with 785 nm laser beam. Acquisition was 15 accumulations of 15 s integrations (225 s total integration time per sample). The spectra were normalized at 1004 cm^−1^ (phenylalanine). The chip was placed on the piezo-stage of the LTRS system and the cells were loaded into the micro-chamber. The cells were optically trapped approximately 20 μm above the glass-liquid interface and spectrographed. Laser tweezer Raman spectroscopy (LTRS) from *E. coli* cells was performed on a maximum of five trapped cells for a single Raman measurement [24]. The assessment of the trapped cell number was based empirically on our observations of the numbers of bacterial cells escaping the trap after the trapping beam was blocked. The effectiveness of optical trapping is influenced by the numerical aperture (NA) of the used microscope objective. This, in turn, defines the maximal size of the Airy disk for a given wavelength, which served us as an approximation of the Gaussian beam waist diameter. The calculated size of the Airy disk was 800 nm in our LTRS setup. The full axial extent (depth) z of the excitation region was calculated to be approximately 4 μm. This value is comparable with the diffraction limit expected for focusing λ = 785 nm light with an NA = 1.2 microscope objective in water. The full lateral extent (width) of the Raman excitation region reaches near the diffraction-limited value Δx = 1.22λ/NA ~ 0.8 μm. The cells were observed by a standard CCD camera through the flipping mirror FM (see Figure 1). During the acquisition of the Raman spectrum, the flipping mirror FM was flipped down and the sample illumination was switched off.

### 2.7. Raman Spectroscopic Characterization of E. coli in the Bulk with 532 nm Excitation

Additional Raman spectroscopic measurements without optical trapping were realized with Renishaw In Via Raman micro spectrometer with 1800 gr/mm diffraction grating, excitation at 532 nm, 100% power (approx. 150 mW at the sample plane), microscope objective 20×, 0.40 NA, NPlanEpi (Leica Microsystems GmbH, Wetzlar, Germany), and 30 accumulations of 1 s for each spectrum. The full lateral extent (width) of the excitation region reached the diffraction-limited value Δx = 1.22λ/NA ~ 1.6 μm. Cells of *E. coli* 683 were prepared as described in Section 2.6. The cell pellet was used to record bulk Raman spectra, which served as a reference to the LTRS experiment.

### 2.8. Processing and Analysis of Raman Spectral Data

In order to extract quantitative information from the acquired spectra, which contain fluorescence along with the Raman signal, we adopted the high-pass signal filter (Rolling Circle Filter—RCF) [32] to separate narrow Raman spectral peaks from the wide spectral background. With an appropriate choice of the filter parameters (filter width and number of filter passes), the background can be effectively removed with no significant distortion of the signal peaks. We kept the same filter parameters for all the measurements presented in this paper. Principal component analysis (PCA) was used for analysis of the obtained Raman spectra. The PCA analysis and RCF were both realized via a homebuilt Raman analysis toolkit based on Matlab (MathWorks, Natick, MA, USA).

## 3. Results and Discussion

### 3.1. Optical Trapping in Microfluidic Environment

**Figure 4:**
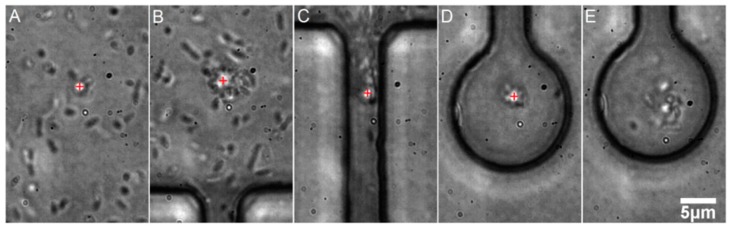
Demonstration of optical trapping and transport of multiple *E. coli* cells from the main microfluidic channel into the micro-chamber. The position of the optical trap is visible as a bright spot near the centers of the images (**A**–**D**) and it is also marked by a red plus sign for clarity. Scale bar (for all panels): 5 μm. (**A**) The optical trap is switched on and a few bacteria are trapped almost immediately; (**B**) The microscope table is operated so that the optical trap is moved towards the neck, dragging with it a swarm of bacterial cells; (**C**) The optical trap passes through the narrow neck, losing some of the trapped cells in the process; (**D**) The optical trap is in the micro-chamber and it contains several cells. (**E**) The optical trap is switched off and the cells disperse in the chamber. It is possible to regulate the amount of trapped bacteria by a proper dilution of the culture in the main channel. We were able to easily load individual bacteria into separate chambers (see Figure 5).

**Figure 5:**
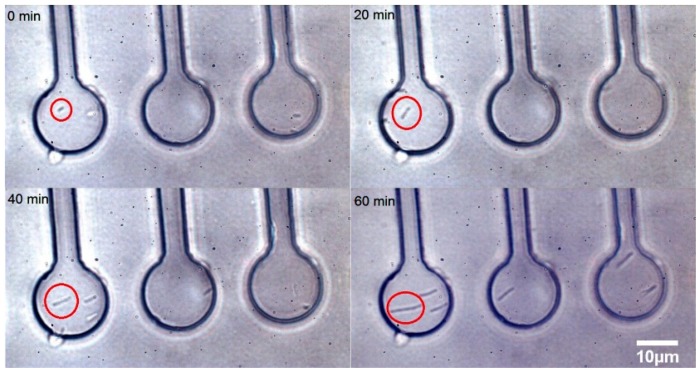
A time-lapse sequence of growing *E. coli* cells is shown in micro-chambers in the presence of 2 mg/L cefotaxime in CASO medium introduced by a syringe pump into the CASO medium running through the microfluidic chip. The red circle shows an individual bacterium elongating over time. The time of cultivation in minutes is given for each quadrant in the top left corner. These bacterial cells were individually loaded into the chambers by optical tweezers. The cells became progressively longer over time in response to the cefotaxime treatment. The red circled bacterium has elongated about 5 times during the 60 min. of microfluidic cultivation. Scale bar (for all panels): 10 μm.

We transported the bacterial cells with optical tweezers into the chambers (see Figure 4). Effectiveness of single particle micromanipulation depended on the concentration of the particles in the channel. Optimal single cell micromanipulation was effective only in highly diluted cell suspensions (see Figure 4 and Figure 5).

### 3.2. Time Lapse Observation of E. coli Growth in Microchambers under Antibiotic Stress

The micro-chamber chip design was used for time-lapse visual and spectroscopic observations of individual cells in a similar manner as in our previous experiments [19] (see Figure 5). The cells were loaded in the microfluidic chambers and the chip was perfused with CASO broth containing 2 mg/L cefotaxime. The cells have elongated about five times during the 60 min. of microfluidic cultivation. This phenomenon was observed previously [33]. Some cephalosporin antibiotics exhibit this effect in a certain range of concentrations since they impair the process of cell division in the sensitive cells [33]. The relatively shallow chambers allowed excellent microscopic observation by keeping the cells near the focal plane. However, the strong Raman signal from the PDMS chip did not allow for successful LTRS in these chambers. This was resolved primarily by using glass cover slips for both the top and bottom surfaces of the chamber and by using deeper chambers to avoid the excessive Raman background from the glass.

### 3.3. Experiments with LTRS of E. coli Cells with 785 nm Wavelength for Trapping and Raman Excitation

**Figure 6:**
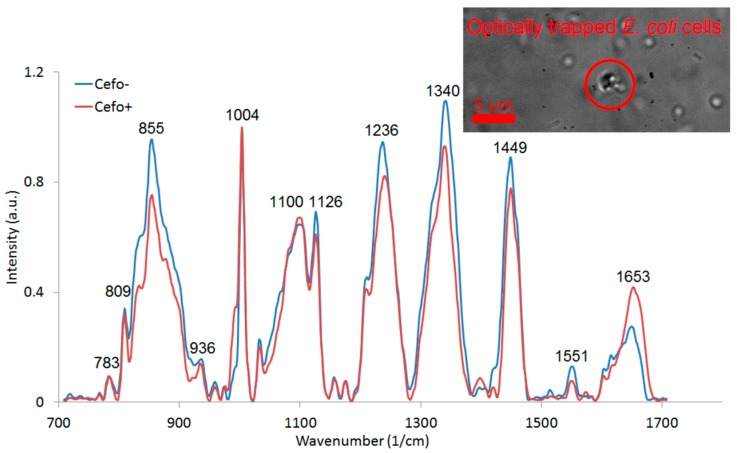
Raman spectra of optically trapped *E. coli* cells cultivated with (+) and without (−) cefotaxime added to the medium. Each spectrum was averaged from 16 (+) and 9 (−) spectra. The spectra show several peaks typical for bacteria. All the major peaks were identified (see Table 1). The inset shows a bright field image of the trapped bacteria prepared for spectroscopic measurement. The red circle defines the optical trap location.

**Figure 7:**
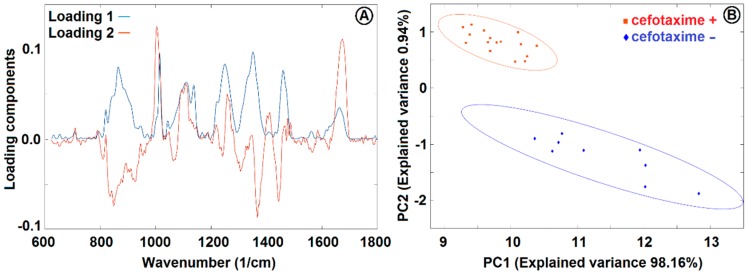
PCA loadings (**A**) and PCA analysis (**B**) of *E. coli* cultivated in CASO broth with and without 2 mg/L cefotaxime added to the medium. See Figure 6 for the Raman spectra and Section 2.6 for sample treatment details. PC1 and PC2 were used for discrimination between the cells with (+) and without (−) cefotaxime. The ellipsoids represent a 95% probability level.

We collected Raman spectra of the optically trapped *E. coli* cultivated for two hours by shaking at 37 °C in CASO broth with (+) and without (−) 2 mg/L cefotaxime added to the medium (see Figure 6). The peaks at 855 cm^−1^, 1126 cm^−1^, 1236 cm^−1^, 1340 cm^−1^, 1449 cm^−1^, and 1551 cm^−1^ decreased with exposition to cefotaxime while the peaks at 1100 cm^−1^ and 1653 cm^−1^ increased with cefotaxime present. We identified all the major peaks and compared their wavenumbers to Reference [11] (see Table 1). We tried to discriminate between the (+) and (−) group with the PCA method. The PCA from the spectra of *E. coli* presented on Figure 6 is depicted on Figure 7. The difference between the (+) and (−) group was highly statistically significant. These data are not fully comparable to the Raman measurements of *E. coli* at 532 nm since the relative peak intensities are rather different with the two excitation wavelengths. The reasons for the observed differences are further discussed in Section 3.4.

### 3.4. Raman Microspectroscopy of E. coli Cells with 532 nm Excitation

**Figure 8:**
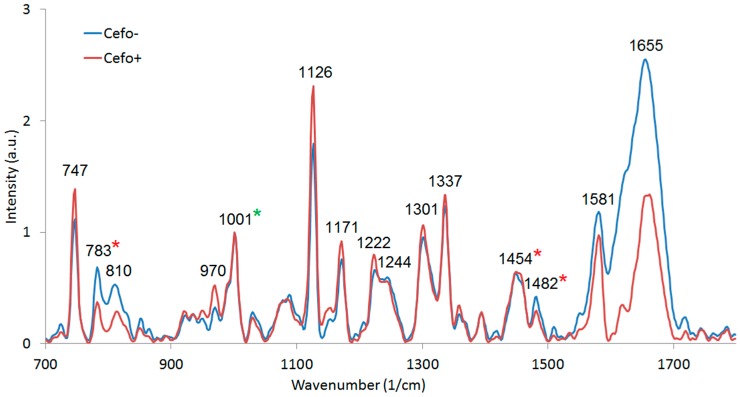
Raman spectra of *E. coli* cells cultivated for three hours in CASO broth with (+) and without (−) cefotaxime and washed with PBS. Averaged from 10 (+) and 6 (−) spectra. Measured at Renishaw In Via with excitation at 532 nm, 100% power, 20× objective and 30 s integration, normalized at 1001 cm^−1^. The normalization peak was highlighted in the spectrum by a green asterisk (*). Red asterisks (*) denote the peaks which were selected for further analysis.

**Figure 9:**
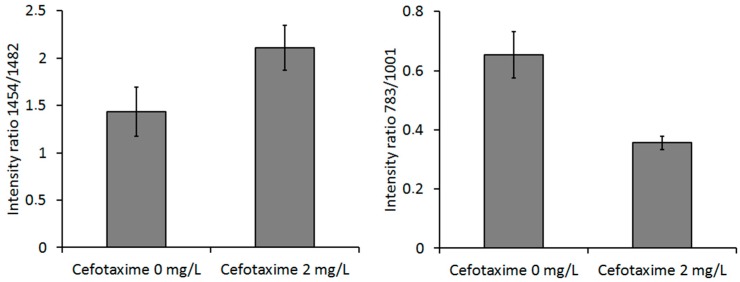
Ratios of Raman peaks for cells cultivated with (2 mg/L) and without (0 mg/L) cefotaxime: 1454/1482 cm^−1^ and 783/1001 cm^−1^. The differences in peak ratios for the experimental and control group were statistically significant. The error bars represent 2 SD.

**Figure 10:**
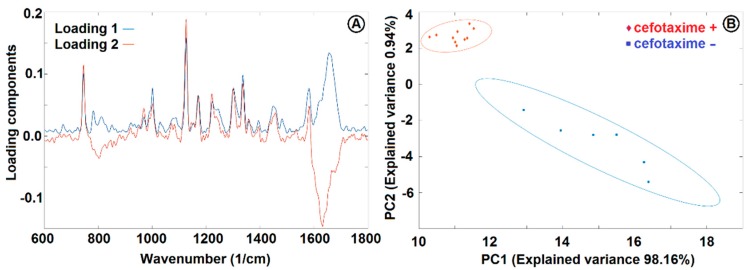
PCA loadings (**A**) and PCA analysis (**B**) of *E. coli* cultivated in CASO broth with and without 2 mg/L cefotaxime added to the medium. See Figure 8 for the Raman spectra and Section 2.7 for sample treatment details. PC1 and PC2 were used for discrimination between the cells with (+) and without (−) cefotaxime. The ellipsoids represent 95% probability level.

We used commercial Raman microspectrometer Renishaw In Via to obtain spectra from *E. coli* cells cultivated in CASO broth with (+) and without (−) cefotaxime with excitation wavelength 532 nm (see Figure 8). We identified the dominant peaks (see Table 1). The spectrum of pure *E. coli* samples includes the peaks around 1458 cm^−1^ and 1485 cm^−1^ (in our case, this was precisely 1454 cm^−1^ and 1482 cm^−1^), which were identified by Kirchhoff et al. [16] as a promising indicator of drug induced changes in *E. coli*. We can see that our results agree with these findings. The 1482 cm^−1^ peak intensity tends to decrease with the presence of cefotaxime relative to the 1454 cm^−1^ peak. Additionally, we have identified in our data and those of Kirchhoff et al. [16] that peak intensity at 783 cm^−1^ invariably decreased in the presence of the antibiotic relatively to the 1001 cm^−1^ signal of phenylalanine. The bar graphs representing the ratios of these peaks are depicted in Figure 9. We further supported our findings with PCA analysis (see Figure 10). PCA analysis was capable of resolving the cells grown with (+) and without (−) cefotaxime with high reliability.

We used the 532 nm excitation wavelength for measuring at Renishaw InVia since the 785 nm excitation beam in combination with the objective used in Renishaw InVia offered too low a power density in the focal area for excitation of a sufficient signal from bacteria without simultaneous trapping. We overcame the problem with low Raman response at 785 nm by using 532 nm excitation because the intensity of the Raman signal (I_RS_) is dependent on the excitation wavelength (λ) by relation: I_RS_ = 1/λ^4^ [34]. Conversely, the LTRS at 532 nm was not realized because of expected heating and photo damage of the studied cells. Moreover, the comparison of Raman spectra obtained from different setups and wavelengths is useful for determining and verification of the optimal analytical strategy for various samples. We also exploited the fact that the shorter excitation wavelength (532 nm) offers higher spectral resolution in otherwise identical conditions. The difference between the spectra obtained in our experiments with different excitation wavelengths is caused by several factors. These factors include near resonance of certain peaks at different wavelengths, the necessity of background removal, and the resulting artifacts (especially for 785 nm where there was higher glass fluorescence), and usage of various microscope objectives, optical elements, and detectors in general. All these factors can influence the relative peak intensity. We did not attempt to quantify these differences due to the limited practical value of the outcome.

We found most of the major Raman bands present in both variants of Raman spectra. Nevertheless, in the Raman spectra obtained with 532 nm excitation wavelength, we have observed some additional Raman peaks. A Raman peak at 747 cm^−1^ was observed previously in bacterial cells, but no specific vibration was associated with it, according to the best and most recent knowledge of the authors [35]. A Raman peak observed at 970 cm^−1^ was found to be similar to 961 cm^−1^ peak representing C–C or C–C–N vibrations in proteins of *E. coli* [36]. Raman peaks close to 1171 cm^−1^ and 1301 cm^−1^ were observed previously in *E. coli* at 1179 cm^−1^ and at 1307 cm^−1^ and associated with vibrations of T and G bases and vibrations of the A base of DNA, respectively [8]. A Raman peak at 1482 cm^−1^ was observed previously [16], but no chemical bond was associated with it, according to the best knowledge of the authors. A Raman peak close to 1581 cm^−1^ was observed previously (at 1586 cm^−1^) and ascribed to vibrations of nucleic acids [36].

## 4. Conclusions

The optical trap and a micro-chamber based on the opto-fluidic system allowed us to effectively isolate the individual bacterial cells of *E. coli* and observe the changes of morphology induced by cephalosporin antibiotic cefotaxime. The system proved to be the ideal combination for simple non-contact micromanipulation of individual cells and their cultivation in a highly controlled environment with the possibility of time-lapse recording of their morphology and development. Based on Raman spectra of optically trapped cells of *E. coli*, we were able to discriminate by PCA between the cells stressed by cefotaxime and the control cultivated in pure CASO broth. We also identified several peaks that changed their magnitude with varying exposure of the cells to cefotaxime. These measurements were realized with 785 nm Raman excitation and trapping wavelength. Raman micro-spectroscopy of bacterial samples at 532 nm provided us with spectra that are complementary to the measurements at 785 nm. These data independently support the finding of Kirchhoff et al. [16] that the ratio of the peaks at 1458 cm^−1^ and 1485 cm^−1^ changes with drug concentration in the medium. We identified and assigned all the major Raman peaks typical for *E. coli* according to a reference [11]. The intensity of peaks and its relative intensity changes were different in the spectra recorded at 785 nm and 532 nm excitation.

We present this work as a proof of principle that our approach combining microfluidic chambers with LTRS provides a solid opto-fluidic platform for single cell manipulation and analysis by optical microscopy and Raman spectroscopy. In order to design a novel microfluidic chip for bacterial separation and identification from different body fluids such as sputum, blood, or urine, we will exploit LTRS in connection with different microfluidic techniques based on centrifugal force, dielectrophoresis, microfiltration, flow-focusing, and surface acoustic wave to sort and cultivate cells in micro-chambers. We are aiming for an advanced connection of microfluidics and optical trapping for analysis of bacteria, which would enable fast and accurate determination of bacterial sepsis.

## Figures and Tables

**Table 1:** Raman peaks of *E. coli* cells and their assignments.

Wavenumber (1/cm) ^1^	Assignment	Wavenumber (1/cm)	Assignment
728 (719, 723)	Adenine	1095 (1100, 1094)	DNA: OPO^−^
783 (783, 783)	Nucleic acids (C, T)	1126 (1126, 1126)	C–N, C, T
813 (809, 810)	Tyrosine	1257 (1236, 1244)	Amide III
857 (855, 853)	Tyrosine	1340 (1340, 1337)	Nucleic acids (A, G)
936 (936, 934)	DNA backbone	1453 (1449, 1454)	C–H_2_ def., lipids
1004 (1004, 1001)	Phenylalanine	1660 (1653, 1655)	Amide I

^1^ Wavenumbers from [11] (633 nm excitation) are presented first (black). The numbers in bracket represent our measurements taken at 785 nm (red) and 532 nm (green) excitation wavelength.
